# An Innovative Reconstruction Technique for Proximal Tibia Grade 3 Giant Cell Tumor: The Sky Roof Reconstruction Approach

**DOI:** 10.7759/cureus.89520

**Published:** 2025-08-06

**Authors:** Sandeep Kumar Yadav, VImal Prakash, Rajesh Kumar Rajnish, Prabodh Kantiwal, Abhay Elhence

**Affiliations:** 1 Orthopedics, All India Institute of Medical Sciences, Jodhpur, Jodhpur, IND

**Keywords:** autologous iliac bone grafting, bone tumour and limb salvage, giant cell tumour of bone, onlay grafting technique, reconstruction hip and knee surgery

## Abstract

Giant cell tumor (GCT) of the bone, although benign, demonstrates local aggressiveness, a potential for recurrence, and, in rare instances, malignant transformation. Functional preservation is crucial in cases involving the articular surface, often utilizing the Sandwich Technique. We propose an enhanced reconstruction method using the inner table of the iliac crest in a reverse fashion, offering a more anatomically contoured proximal tibial plateau and reducing donor site morbidity compared to tricortical iliac crest grafting. This technique ensures that the marrow and cancellous bone facing the articular cartilage provide essential nourishment for articular cartilage, preventing early joint arthritis.

## Introduction

Giant cell tumor (GCT) of bone is a benign but locally aggressive neoplasm, characterized by a tendency to recur and, in rare cases, undergo malignant transformation. In cases involving the articular surface, functional preservation is of paramount importance, and the Sandwich Technique is often employed. This technique involves extended curettage of the lesion, subchondral support using morselized bone graft pieces, and filling the resulting cavity with polymethylmethacrylate (PMMA) bone cement, bone substitutes, or bone graft [1,2]. Studies have documented instances wherein tricortical iliac crest bone grafting is utilized for subchondral support due to ease of access [2,3]. We propose a novel technique using the inner table of the iliac crest in a reverse fashion. This innovative approach not only provides a more anatomically contoured proximal tibial plateau compared to morselized graft pieces but also minimizes donor site morbidity compared to tricortical iliac crest grafting. Importantly, the marrow and cancellous bone facing the articular cartilage provide nourishment to the cartilage, potentially preventing early joint arthritis.

## Technical report

Preoperative evaluation

**Figure 1:**
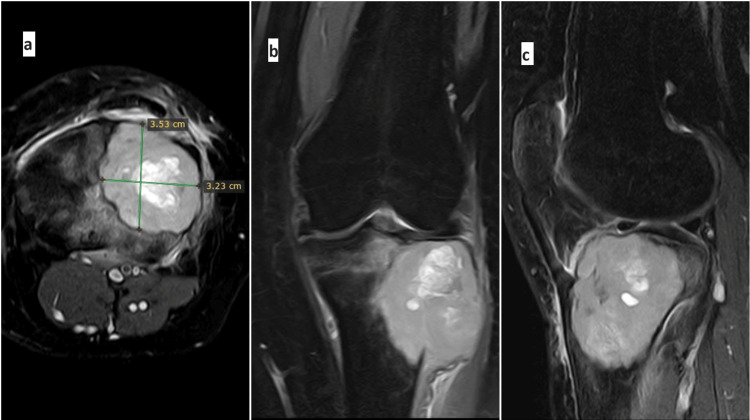
Preoperative evaluation and planning a: The size of the subchondral bone defect was measured in axial T2 images. (This can be done with CT axial images also.) A subchondral bone defect measuring 3.5 x 3.23 cm was found, which is the expected size of the inner column iliac crest graft to be taken. Exact measurements of the required graft will be taken intraoperatively after curettage of the lesion. b,c: Coronal and sagittal T2 MRI images showing the extent of the lesion CT: computed tomography; MRI: magnetic resonance imaging

Thorough history-taking is imperative, with emphasis on the duration of symptoms. Comprehensive knee examinations, both general and local, are essential. This includes the evaluation of swelling, identification of the site of tenderness, and assessment of the knee range of motion. To establish a provisional diagnosis and assess tumor characteristics accurately, imaging studies such as knee and leg X-rays, CT, and MRI should be performed. CT and MRI are crucial in determining the size and extent of the lesion, evaluating cortical breach, and measuring the subchondral bone loss that requires reconstruction (Figure 1).

A confirmatory diagnosis was obtained through a core needle biopsy.

Surgical approach 

**Figure 2:**
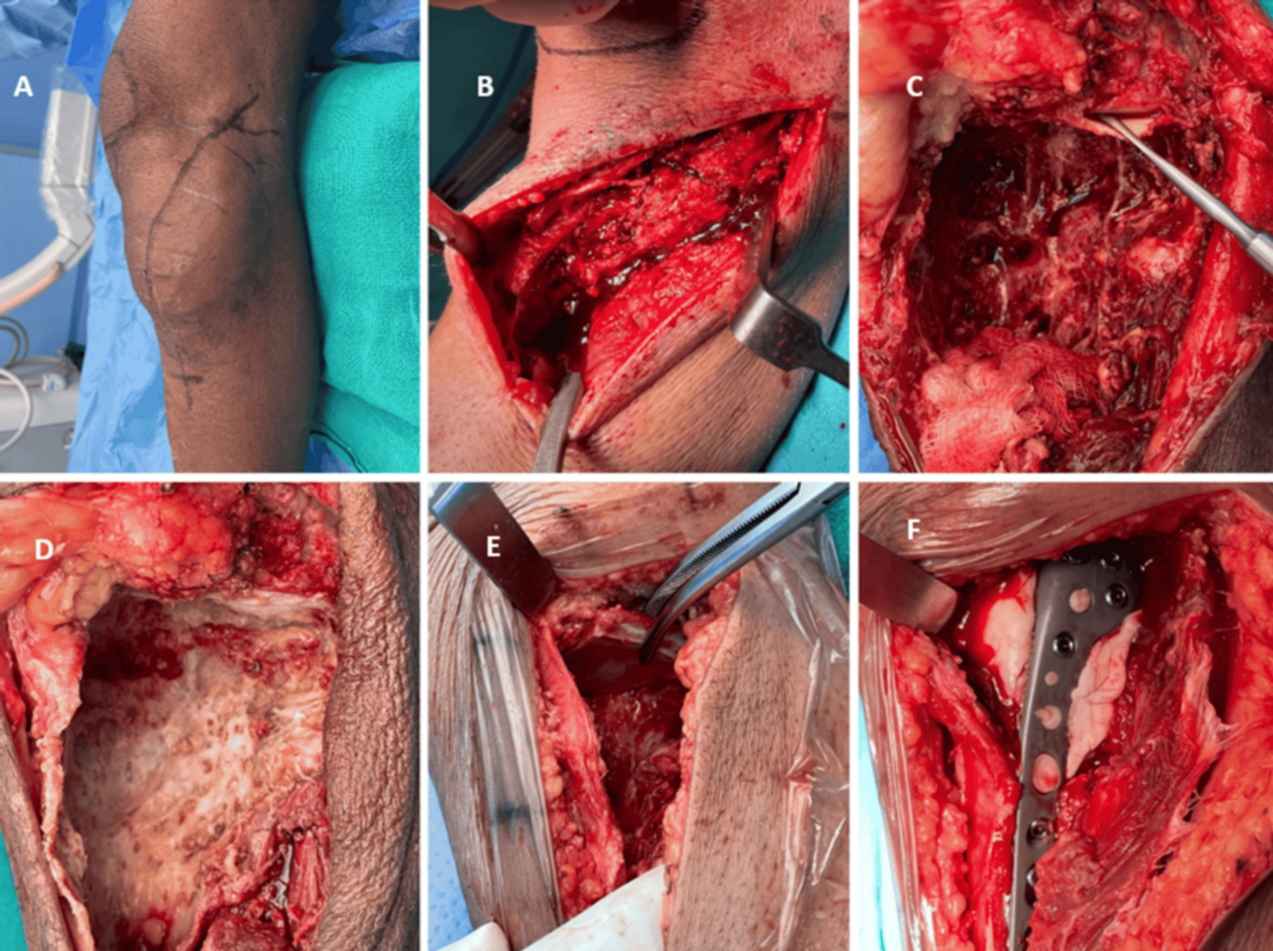
Intraoperative images A and B: Standard anterolateral approach to the proximal tibia. C: Proximal tibia after extended curettage with exposed undersurface of articular cartilage. D: Cavity after extended curettage. E: Positioning of the harvested iliac crest graft in the cancellous inner part in the upside fashion to fill the measured defect of subchondral bone. F: Filling the remaining cavity with bone cement and fixation with the plate

A standard anterolateral approach to the knee and proximal tibia is employed. An anterolateral curved incision is made, extending from the lateral aspect of the patella to the anterolateral aspect of the proximal tibia. The deep fascia anterior to the iliotibial tract is incised, and the proximal attachment of the tibialis anterior muscle is released. If the cortex is intact, a cortical window is carefully created anterolaterally (Figure 2).

Curettage and high-speed burring

Curettage is performed using bone curettes of various sizes. This is followed by the use of a high-speed burr to extend the curettage beyond the tumor margin, with caution taken to avoid damage to the articular cartilage. The cavity is then thoroughly washed with hydrogen peroxide and saline. The dimensions and shape of the resulting subchondral bone defect are measured to determine the size of the required iliac crest graft.

Inner column iliac crest bone graft harvesting

An inner table iliac crest bone graft is harvested from the ipsilateral side. The size of the graft is based on the measurements obtained from preoperative CT and MRI. Using a combination of an oscillating saw and osteotome, the inner table graft is harvested and shaped. The graft must be large enough to adequately reconstruct the subchondral bone defect.

Reconstruction of the proximal tibia

**Figure 3:**
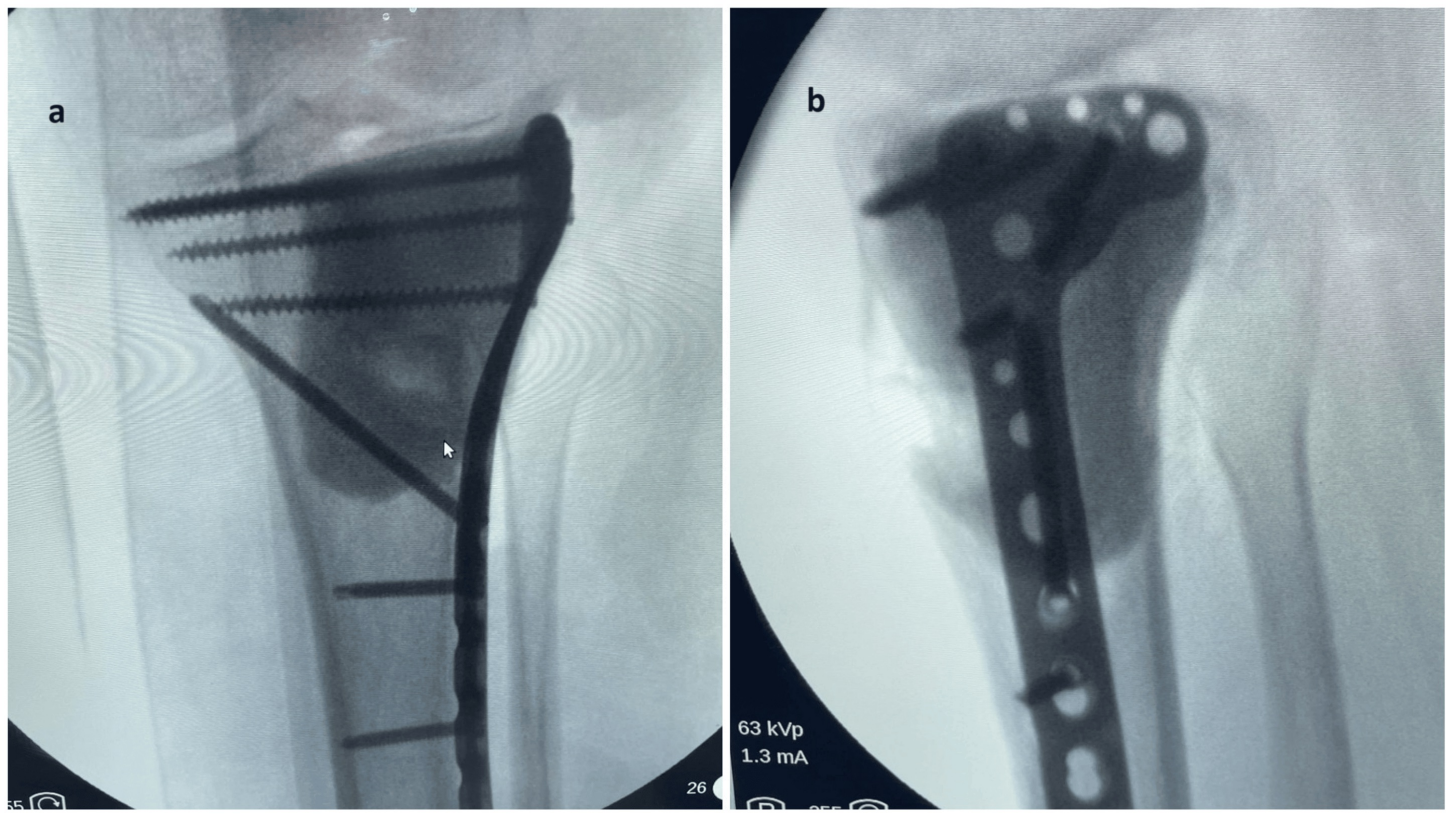
Intraoperative fluroscopy images Intraoperative radiographs after the completion of the procedure (a: anteroposterior view; b: lateral view)

The harvested graft is trimmed using a bone nibbler to achieve a shape that conforms anatomically to the defect in the subchondral region. It is placed beneath the articular cartilage with the contoured cancellous surface facing upward, directly supporting the cartilage and restoring the anatomical contour of the proximal tibia. A suitable anterolateral raft plate is applied to provide mechanical stabilization (Figure 3). Gel foam is placed beneath the graft, and the remaining cavity is filled with bone cement. A drain is inserted, and the wound is closed in layers.

Postoperative follow-up and rehabilitation

The operated leg is immobilized in a below-knee splint. Immediate postoperative X-rays are obtained. The drain is removed after 24 hours. The splint and sutures are removed at two weeks postoperatively. Subsequently, the patient begins a range of motion exercises for the knee, along with quadriceps and hamstring strengthening exercises, with non-weight bearing for the first six weeks. Partial weight bearing as tolerated is allowed during the second six weeks, with full weight bearing beginning three months postoperatively.

**Figure 4:**
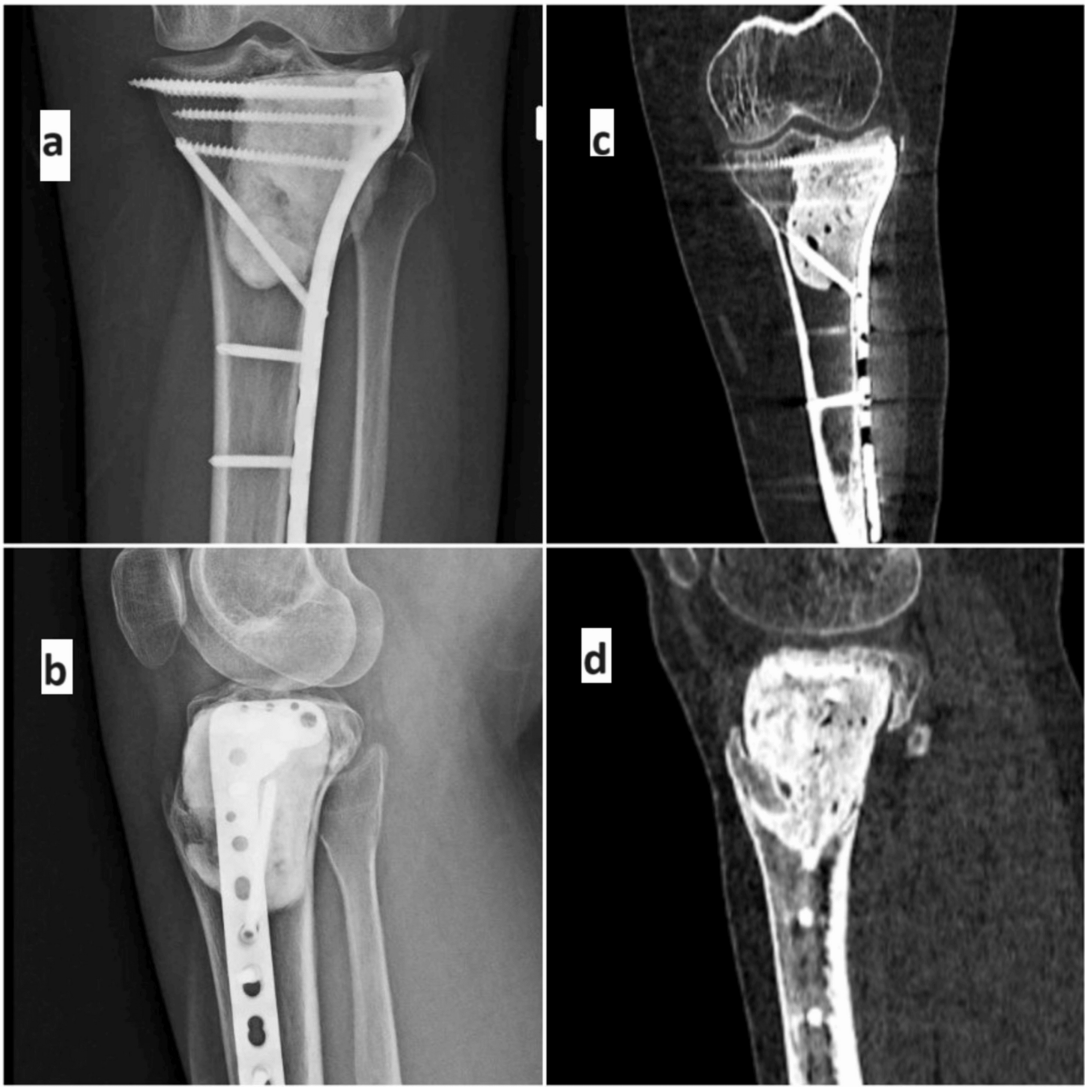
Follow-up radiographs and CT scan Three-year follow-up imaging (a, b: plain radiographs, anteroposterior, and lateral projections, respectively, showing anatomically contoured articular surface. c, d: CT coronal and sagittal images showing well-incorporated graft and smooth articular surface.) CT: computed tomography

Regular follow-up should include clinical evaluations, radiographs, and CT scans to assess graft union, joint surface restoration, and detect any tumor recurrence. Radiographs are recommended every six months for the first two years and annually thereafter for five years. Our patient was able to meet all functional demands and had a full, painless range of knee motion with radiological evidence of healing at the three-year follow-up (Figure 4).

## Discussion

GCTs are typically benign neoplasms that occur in the epi-metaphyseal region of skeletally mature patients, constituting approximately 5% of all primary bone tumors. They commonly manifest in long bones but can also affect the axial skeleton and small bones of the hands and feet, with the distal femur and proximal tibia being the most frequently involved sites [3-5]. Treatment options for GCTs around the knee include curettage alone, curettage with adjuvant therapies (e.g., liquid nitrogen, hydrogen peroxide, phenol, argon laser photocoagulation), bone cement, bone graft, and marginal or wide resection followed by reconstruction, arthrodesis, or mega-prosthetic joint replacement. Intralesional curettage alone is associated with a recurrence rate as high as 60%, whereas marginal or wide resection can lead to significant functional disability. Preservation of joint function is a notable advantage of intralesional curettage over wide resection. Radiotherapy is reserved for spinal, sacral, or aggressive lesions when complete excision or curettage is not feasible due to anatomical or medical constraints [3].

The Sandwich Technique provides structural and biological support following curettage. It involves creating a wide cortical window for thorough tumor curettage, often aided by adjuvants such as a high-speed burr, phenol, hydrogen peroxide, or liquid nitrogen. After tumor removal, morselized structural bone graft (3-5 mm pieces) is packed subchondrally and covered with gel foam, followed by filling the remaining cavity with PMMA bone cement-the thermal properties of cement aid in eliminating residual tumor cells. The graft under the subchondral bone preserves the articular surface and prevents early degeneration, while gel foam provides a thermal barrier between cement and cartilage [1].

However, the conventional Sandwich Technique has limitations, including the potential for articular cartilage necrosis due to impaired subchondral nutrition, lack of structural integrity from morselized grafts, and poor integration of these graft fragments. To overcome these drawbacks, we propose using an inner table iliac crest autograft with the cancellous surface facing upward, toward the articular cartilage. The cancellous architecture of the inner table allows it to be contoured to match the anatomy of the proximal tibial plateau and provides superior subchondral support [1,2].

Recent studies support the role of subchondral bone marrow in articular cartilage nutrition. According to Wang et al., articular cartilage receives nutrition primarily via synovial diffusion and secondarily from subchondral marrow. Their experimental findings using histological, immunohistochemical, and molecular analyses demonstrated that impairment of subchondral marrow nutrition leads to early cartilage degeneration [6]. Hence, our method ensures that the cancellous surface of the graft remains in contact with the articular cartilage, potentially supporting its nourishment and longevity.

Although tricortical iliac crest grafts have been used for subchondral reconstruction, harvesting them may result in significant complications, such as hematoma, herniation of abdominal contents, lateral femoral cutaneous nerve injury, chronic iliac pain, limping, and even iliac crest fracture [7]. Our technique minimizes these complications by harvesting only from the inner table of the iliac crest, leaving the outer table intact. This approach preserves the iliac contour and reduces postoperative donor site morbidity [8].

## Conclusions

The use of an inner table iliac crest autograft in reverse orientation provides an effective and biologically favorable approach for reconstructing subchondral bone defects following curettage of proximal tibial GCTs. This technique facilitates anatomical restoration of the joint surface, ensures structural stability, and offers nutritional support to the overlying articular cartilage, potentially reducing the risk of early osteoarthritis. Compared to conventional morselized or tricortical grafts, it minimizes donor site morbidity while enhancing graft integration. At a three-year follow-up, our patient demonstrated excellent functional and radiological outcomes. This method offers a promising alternative for joint-preserving procedures in periarticular tumors and merits broader clinical adoption and long-term outcome evaluation.
